# 1-(4-Hydr­oxy-2-methyl-1,1-dioxo-2*H*-1,2-benzothia­zin-3-yl)ethanone

**DOI:** 10.1107/S1600536808007721

**Published:** 2008-04-02

**Authors:** Matloob Ahmad, Hamid Latif Siddiqui, Muhammad Zia-ur-Rehman, Muhammad Irfan Ashiq, Graham John Tizzard

**Affiliations:** a Institute of Chemistry, University of the Punjab, Lahore-54590, Pakistan; b Applied Chemistry Research Centre, PCSIR Laboratories Complex, Lahore-54600, Pakistan; c School of Chemistry, University of Southampton, England

## Abstract

In the title compound, C_11_H_11_NO_4_S, the thia­zine ring adopts a distorted half-chair conformation. The enolic H atom is involved in an intra­molecular O—H⋯O hydrogen bond, forming a six-membered ring. Mol­ecules are linked through weak inter­molecular C—H⋯O hydrogen bonds, resulting in chains lying along the *b* axis.

## Related literature

For related literature, see: Bihovsky *et al.* (2004 ▶); Fabiola *et al.* (1998 ▶); Golič & Leban (1987 ▶); Zia-ur-Rehman *et al.* (2005 ▶, 2006 ▶, 2007 ▶); Turck *et al.* (1996 ▶).
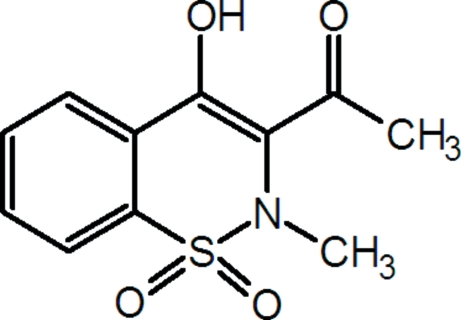


## Experimental

### 

#### Crystal data

C_11_H_11_NO_4_S
*M* *_r_* = 253.27
Triclinic, 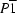

*a* = 6.8523 (1) Å
*b* = 8.3222 (2) Å
*c* = 10.4880 (2) Å
α = 72.1321 (11)°
β = 77.9619 (12)°
γ = 80.0360 (12)°
*V* = 552.89 (2) Å^3^
*Z* = 2
Mo *K*α radiation
μ = 0.29 mm^−1^
*T* = 120 (2) K
0.40 × 0.20 × 0.14 mm

#### Data collection

Bruker–Nonius KappaCCD diffractometer
Absorption correction: multi-scan (*SADABS*; Sheldrick, 2007 ▶) *T*_min_ = 0.891, *T*_max_ = 0.960
12691 measured reflections
2529 independent reflections
2248 reflections with *I* > 2σ(*I*)
*R*_int_ = 0.033

#### Refinement

*R*[*F* ^2^ > 2σ(*F* ^2^)] = 0.034
*wR*(*F* ^2^) = 0.092
*S* = 1.11
2529 reflections
157 parameters
H-atom parameters constrained
Δρ_max_ = 0.31 e Å^−3^
Δρ_min_ = −0.53 e Å^−3^

### 

Data collection: *COLLECT* (Nonius, 1998 ▶); cell refinement: *DENZO* (Otwinowski & Minor, 1997 ▶); data reduction: *DENZO* and *COLLECT*; program(s) used to solve structure: *SHELXS97* (Sheldrick, 2008 ▶); program(s) used to refine structure: *SHELXL97* (Sheldrick, 2008 ▶); molecular graphics: *CAMERON* (Pearce & Watkin, 1993 ▶); software used to prepare material for publication: *WinGX* (Farrugia, 1999 ▶).

## Supplementary Material

Crystal structure: contains datablocks I, global. DOI: 10.1107/S1600536808007721/pv2072sup1.cif

Structure factors: contains datablocks I. DOI: 10.1107/S1600536808007721/pv2072Isup2.hkl

Additional supplementary materials:  crystallographic information; 3D view; checkCIF report

## Figures and Tables

**Table 1:** Hydrogen-bond geometry (Å, °)

*D*—H⋯*A*	*D*—H	H⋯*A*	*D*⋯*A*	*D*—H⋯*A*
O3—H3*A*⋯O4	0.84	1.78	2.525 (2)	146
C4—H4⋯O2^i^	0.95	2.36	3.193 (2)	146

Symmetry code: (i) 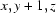.

**Fig. 1.**
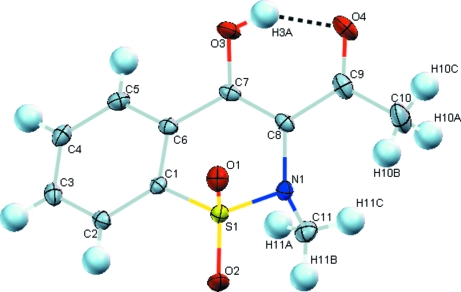
The molecular structure of (I), showing displacement ellipsoids at the 50% probability level for non-H atoms; dashed lines denote hydrogen bonds.

**Fig. 2.**
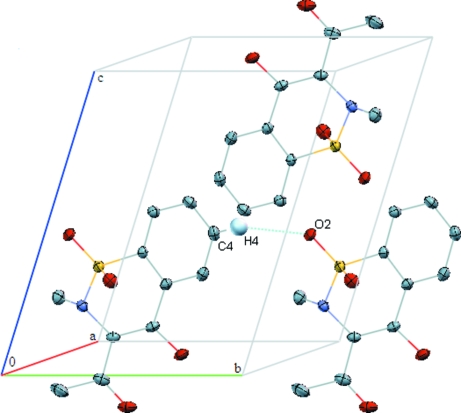
Unit cell packing of (I), showing intermolecular H-bonds resulting in the chains of molecules lying along the b axis; H atoms not involved in H-bonding have been omitted.

## supplementary crystallographic information

### Comment

In order to discover new useful therapeutic agents, many new compounds are continuously being synthesized. Benzothiazine dioxides and their derivatives are reported to possess numerous types of biological activities. Owing to their applications as non-steroidal anti-inflammatory compounds (Turck *et al*., 1996), considerable attention has been given to 1,2-benzothiazine 1,1-dioxides and their precursor intermediates (Golič & Leban, 1987). Various 1,2-benzothiazines derivatives are also known as potent calpain I inhibitors (Bihovsky *et al.*, 2004), while benzothiaine-3-yl-quinazolin-4-ones showed marked activity against *Bacillus subtilis *(Zia-ur-Rehman *et al.*, 2006). As part of a research program synthesizing various bioactive benzothiazines (Zia-ur-Rehman *et al.*, 2005, 2006), we herein report the crystal structure of the title compound, (I).

In the molecule of the title compound (Fig. 1), the thiazine ring exhibits a distorted half-chair conformation with S1/C1/C6/C7 atoms lying in a plane and N1 showing significant departure from the plane due to its pyramidal geometry projecting the methyl group approximately perpendicular to the ring; the deviations of N1 and C8 from the least square plane being -0.895 (2) and -0.413 (3) Å, respectively. Like other 1,2-benzothiazine 1,1-dioxide derivatives (Fabiola *et al.*, 1998; Zia-ur-Rehman *et al.*, 2007), the enolic hydrogen on O3 is involved in intramolecular hydrogen bonding (Table 1). Also, C7—C8 bond length [1.378 Å] (very close to normal C?C bond; 1.36 Å) indicates a partial double-bond character indicating the dominance of enolic form in the molecule. The C1—S1 bond distance [1.7580 (14) Å] is as expected for typical C(*sp*^2^)—S bond (1.751 Å).

Each molecule is linked to its adjacent one through a hydrogen bond [C4—H4···O2] resulting in a chain of molecules lying along the *b* axis (Table 1 and Fig. 2). Each molecule in the chain is linked with its neighbour through weak slipped π-π interactions at inversion centres; the closest C–C contacts are between C3–C3^i^ separated by 3.316 Å.

### Experimental

A mixture of 1-(4-hydroxy-1,1-dioxido-2*H*-1,2-benzothiazin-3-yl) ethanone (239 mg, 1.0 mmol) dissolved in acetone (10 ml), aqueous NaOH (3 ml, 5%) and dimethyl sulfate (0.5 ml) was stirred for half an hour followed by careful addition of dilute HCl (5%) to maintain the pH to Congo Red. Precipitates of (I) thus obtained were filtered, washed with water and dried. Colourless crystals were grown by slow evaporation of a solution of (I) in methanol at room temperature.

### Refinement

The hydrogen atoms were included in the refinements in a riding mode with the following constraints: aryl, methyl and hydroxyl C/O—H distances 0.95, 0.98 and 0.84 Å, respectively, and *U*_iso_(H) = 1.2 *U*_eq_(aryl C) and 1.5 *U*_eq_(methyl C and O).

### Crystal data

**Table:**

C_11_H_11_NO_4_S	*Z* = 2
*M**_r_* = 253.27	*F*_000_ = 264
Triclinic, *P*1	*D*_x_ = 1.521 Mg m^−^^3^
Hall symbol: -P 1	Mo *K*α radiation λ = 0.71073 Å
*a* = 6.8523 (1) Å	Cell parameters from 15360 reflections
*b* = 8.3222 (2) Å	θ = 2.9–27.5º
*c* = 10.4880 (2) Å	µ = 0.30 mm^−^^1^
α = 72.1321 (11)º	*T* = 120 (2) K
β = 77.9619 (12)º	Shard, colourless
γ = 80.0360 (12)º	0.40 × 0.20 × 0.14 mm
*V* = 552.892 (19) Å^3^	

### Data collection

**Table:**

Bruker–Nonius CCD camera on κ-goniostat diffractometer	2529 independent reflections
Radiation source: Bruker Nonius FR591 Rotating Anode	2248 reflections with *I* > 2σ(*I*)
Monochromator: graphite	*R*_int_ = 0.033
Detector resolution: 9.091 pixels mm^-1^	θ_max_ = 27.5º
*T* = 120(2) K	θ_min_ = 3.1º
φ & ω scans to fill the asymmetric unit	*h* = −8→8
Absorption correction: multi-scan(SADABS; Sheldrick, 2007)	*k* = −10→10
*T*_min_ = 0.891, *T*_max_ = 0.960	*l* = −13→13
12691 measured reflections	

### Refinement

**Table:**

Refinement on *F*^2^	Secondary atom site location: difference Fourier map
Least-squares matrix: full	Hydrogen site location: inferred from neighbouring sites
*R*[*F*^2^ > 2σ(*F*^2^)] = 0.034	H-atom parameters constrained
*wR*(*F*^2^) = 0.092	*w* = 1/[σ^2^(*F*_o_^2^) + (0.041*P*)^2^ + 0.3066*P*] where *P* = (*F*_o_^2^ + 2*F*_c_^2^)/3
*S* = 1.11	(Δ/σ)_max_ = 0.030
2529 reflections	Δρ_max_ = 0.31 e Å^−^^3^
157 parameters	Δρ_min_ = −0.53 e Å^−^^3^
Primary atom site location: structure-invariant direct methods	Extinction correction: none

### Special details

**Table:**

Experimental. *SADABS* was used to perform the Absorption correction Estimated minimum and maximum transmission: 0.6696 0.7456 The given Tmin and Tmax were generated using the *SHELX* SIZE command
Geometry. All e.s.d.'s (except the e.s.d. in the dihedral angle between two l.s. planes) are estimated using the full covariance matrix. The cell e.s.d.'s are taken into account individually in the estimation of e.s.d.'s in distances, angles and torsion angles; correlations between e.s.d.'s in cell parameters are only used when they are defined by crystal symmetry. An approximate (isotropic) treatment of cell e.s.d.'s is used for estimating e.s.d.'s involving l.s. planes.
Refinement. Refinement of *F*^2^ against ALL reflections. The weighted *R*-factor *wR* and goodness of fit *S* are based on *F*^2^, conventional *R*-factors *R* are based on *F*, with *F* set to zero for negative *F*^2^. The threshold expression of *F*^2^ > σ(*F*^2^) is used only for calculating *R*-factors(gt) *etc*. and is not relevant to the choice of reflections for refinement. *R*-factors based on *F*^2^ are statistically about twice as large as those based on *F*, and *R*- factors based on ALL data will be even larger.

### Fractional atomic coordinates and isotropic or equivalent isotropic displacement parameters (Å^2^)

**Table:**

	*x*	*y*	*z*	*U*_iso_*/*U*_eq_	
C1	0.3007 (2)	0.32223 (18)	0.37041 (14)	0.0135 (3)	
C2	0.2992 (2)	0.31686 (19)	0.50412 (15)	0.0164 (3)	
H2	0.3246	0.2117	0.5702	0.020*	
C3	0.2595 (2)	0.4692 (2)	0.53933 (16)	0.0191 (3)	
H3	0.2575	0.4684	0.6304	0.023*	
C4	0.2228 (2)	0.6220 (2)	0.44168 (16)	0.0199 (3)	
H4	0.1973	0.7252	0.4665	0.024*	
C5	0.2229 (2)	0.62640 (19)	0.30802 (15)	0.0184 (3)	
H5	0.1983	0.7320	0.2422	0.022*	
C6	0.2593 (2)	0.47505 (18)	0.27093 (14)	0.0148 (3)	
C7	0.2558 (2)	0.47385 (19)	0.13150 (14)	0.0153 (3)	
C8	0.2396 (2)	0.32881 (19)	0.09905 (14)	0.0157 (3)	
C9	0.2507 (2)	0.3316 (2)	−0.04098 (16)	0.0204 (3)	
C10	0.2518 (3)	0.1701 (2)	−0.07671 (18)	0.0332 (4)	
H10A	0.1136	0.1532	−0.0764	0.050*	
H10B	0.3113	0.0740	−0.0098	0.050*	
H10C	0.3310	0.1775	−0.1672	0.050*	
C11	0.0024 (2)	0.1379 (2)	0.25625 (16)	0.0210 (3)	
H11A	−0.0710	0.2301	0.2933	0.031*	
H11B	−0.0022	0.0298	0.3279	0.031*	
H11C	−0.0598	0.1321	0.1819	0.031*	
N1	0.21494 (18)	0.17080 (16)	0.20411 (12)	0.0152 (3)	
O1	0.56456 (16)	0.12980 (14)	0.24794 (11)	0.0202 (2)	
O2	0.30103 (17)	−0.00587 (13)	0.42939 (11)	0.0212 (3)	
O3	0.27313 (18)	0.62312 (14)	0.03808 (11)	0.0220 (3)	
H3A	0.2785	0.6110	−0.0392	0.033*	
O4	0.26152 (18)	0.46897 (16)	−0.13289 (11)	0.0264 (3)	
S1	0.36145 (5)	0.13574 (4)	0.31790 (3)	0.01424 (12)	

### Atomic displacement parameters (Å^2^)

**Table:**

	*U*^11^	*U*^22^	*U*^33^	*U*^12^	*U*^13^	*U*^23^
C1	0.0111 (6)	0.0140 (7)	0.0157 (7)	−0.0025 (5)	−0.0013 (5)	−0.0046 (5)
C2	0.0141 (7)	0.0192 (7)	0.0154 (7)	−0.0029 (6)	−0.0034 (5)	−0.0033 (6)
C3	0.0167 (7)	0.0257 (8)	0.0181 (7)	−0.0037 (6)	−0.0031 (6)	−0.0101 (6)
C4	0.0189 (7)	0.0187 (8)	0.0251 (8)	−0.0044 (6)	−0.0014 (6)	−0.0107 (6)
C5	0.0181 (7)	0.0145 (7)	0.0205 (7)	−0.0024 (5)	−0.0011 (6)	−0.0032 (6)
C6	0.0128 (7)	0.0160 (7)	0.0148 (7)	−0.0026 (5)	−0.0010 (5)	−0.0034 (5)
C7	0.0120 (7)	0.0171 (7)	0.0132 (7)	0.0003 (5)	−0.0008 (5)	−0.0009 (5)
C8	0.0135 (7)	0.0193 (7)	0.0129 (7)	0.0005 (5)	−0.0023 (5)	−0.0037 (5)
C9	0.0164 (7)	0.0277 (8)	0.0176 (7)	0.0039 (6)	−0.0049 (6)	−0.0092 (6)
C10	0.0463 (11)	0.0342 (10)	0.0245 (9)	0.0043 (8)	−0.0133 (8)	−0.0164 (7)
C11	0.0152 (7)	0.0230 (8)	0.0249 (8)	−0.0049 (6)	−0.0035 (6)	−0.0056 (6)
N1	0.0144 (6)	0.0165 (6)	0.0157 (6)	−0.0018 (5)	−0.0045 (5)	−0.0050 (5)
O1	0.0138 (5)	0.0234 (6)	0.0246 (6)	0.0025 (4)	−0.0036 (4)	−0.0108 (5)
O2	0.0292 (6)	0.0133 (5)	0.0202 (5)	−0.0044 (4)	−0.0086 (5)	0.0005 (4)
O3	0.0287 (6)	0.0182 (6)	0.0143 (5)	−0.0019 (5)	−0.0022 (5)	0.0009 (4)
O4	0.0300 (6)	0.0322 (7)	0.0140 (5)	0.0020 (5)	−0.0058 (5)	−0.0038 (5)
S1	0.01453 (19)	0.01270 (19)	0.01608 (19)	0.00015 (13)	−0.00467 (13)	−0.00442 (13)

### Geometric parameters (Å, °)

**Table:**

C1—C2	1.387 (2)	C8—C9	1.448 (2)
C1—C6	1.404 (2)	C9—O4	1.251 (2)
C1—S1	1.7580 (14)	C9—C10	1.501 (2)
C2—C3	1.394 (2)	C10—H10A	0.9800
C2—H2	0.9500	C10—H10B	0.9800
C3—C4	1.388 (2)	C10—H10C	0.9800
C3—H3	0.9500	C11—N1	1.4864 (19)
C4—C5	1.391 (2)	C11—H11A	0.9800
C4—H4	0.9500	C11—H11B	0.9800
C5—C6	1.397 (2)	C11—H11C	0.9800
C5—H5	0.9500	N1—S1	1.6438 (12)
C6—C7	1.471 (2)	O1—S1	1.4319 (11)
C7—O3	1.3316 (17)	O2—S1	1.4314 (11)
C7—C8	1.378 (2)	O3—H3A	0.8400
C8—N1	1.4430 (18)		
			
C2—C1—C6	122.15 (13)	O4—C9—C10	119.76 (14)
C2—C1—S1	120.70 (11)	C8—C9—C10	120.31 (15)
C6—C1—S1	117.13 (11)	C9—C10—H10A	109.5
C1—C2—C3	118.54 (14)	C9—C10—H10B	109.5
C1—C2—H2	120.7	H10A—C10—H10B	109.5
C3—C2—H2	120.7	C9—C10—H10C	109.5
C4—C3—C2	120.14 (14)	H10A—C10—H10C	109.5
C4—C3—H3	119.9	H10B—C10—H10C	109.5
C2—C3—H3	119.9	N1—C11—H11A	109.5
C3—C4—C5	121.04 (14)	N1—C11—H11B	109.5
C3—C4—H4	119.5	H11A—C11—H11B	109.5
C5—C4—H4	119.5	N1—C11—H11C	109.5
C4—C5—C6	119.79 (14)	H11A—C11—H11C	109.5
C4—C5—H5	120.1	H11B—C11—H11C	109.5
C6—C5—H5	120.1	C8—N1—C11	114.26 (11)
C5—C6—C1	118.30 (13)	C8—N1—S1	112.75 (10)
C5—C6—C7	121.47 (13)	C11—N1—S1	116.79 (10)
C1—C6—C7	120.23 (13)	C7—O3—H3A	109.5
O3—C7—C8	122.30 (13)	O2—S1—O1	119.39 (7)
O3—C7—C6	114.96 (13)	O2—S1—N1	108.47 (7)
C8—C7—C6	122.73 (13)	O1—S1—N1	107.24 (6)
C7—C8—N1	120.44 (12)	O2—S1—C1	109.27 (7)
C7—C8—C9	120.64 (14)	O1—S1—C1	108.93 (7)
N1—C8—C9	118.91 (13)	N1—S1—C1	102.15 (6)
O4—C9—C8	119.92 (14)		

### Hydrogen-bond geometry (Å, °)

**Table:**

*D*—H···*A*	*D*—H	H···*A*	*D*···*A*	*D*—H···*A*
O3—H3A···O4	0.84	1.78	2.525 (2)	146
C4—H4···O2^i^	0.95	2.36	3.193 (2)	146

Symmetry codes: (i) *x*, *y*+1, *z*.
